# Case of Aneurism of the Heart, with Perforation in the Walls of the Auricle.—Journ. de Med.

**Published:** 1814-11

**Authors:** 


### *For the Medical and Physical Journal.*

### *Case of Aneurism of the Heart, with Perforation in the Walls of the Auricle.*—Journ. de Med.

MR. N., whilst riding at full gallop, felt a pain in the right side of the chest, as if he had received a blow on this part. In the evening his respiration was difficult, but he supped heartily, and slept well. At midnight his sleep was suddenly interrupted by a noise like that made by a saw; he awoke his wife, who laid at his side; she distinctly heard the same noise, but was at a loss to know whence it arose; she got up, and found that as she removed from her husband it diminished, and *vice versâ*; at last they discovered that it was seated in his chest. On applying the hand to the region of the heart, strong pulsations were felt. In this situation he remained many days. The difficulty of breathing increases as he ascends the staircase. He complains of a feeling of anguish in the epigastrium, with vertigo, head-ach, flushings of the face, palpitations, and discovers great uneasiness for his fate.

Being called in consultation at the end of September, I examined him attentively. Striking the right side of the chest, it gave a dull sound throughout its whole extent. The pulse is strong, full, and *without irregularity.* The patient lies either on the back or the sides; if he walks quickly, his respiration is affected. Conceiving this to be an aneurism of the ventricles of the heart, I ordered him to be bled, and leeches to be applied to the anus, with mild aperient remedies. He had pediluvia and baths for the anus. I prescribed for him the digitalis purpurea, with an anti-spasmodic draught, and advised him to abstain from exercise, wine, and spirituous liquors, and to be sparing in his diet. About January a cough arose, which was relieved by ordinary medicines, and he was somewhat better for the first two months after the commencement of this treatment.

About the beginning of April, the oppression at the chest became stronger, he was more extenuated, the lips were of a bluish colour, his cough returned with great distress, and the expectoration was frequently bloody; he could not move without experiencing the most violent palpitation, extreme uneasiness in the chest, and imminent suffocation; he complained of very acute pain along the passage of the carotid, radial, crural, and popliteal, arteries; his pulse was uniform, but weak; and he grew daily worse. In the last week of the same month, he did not get the smallest rest; if his eyeeye-lids closed, and he began to sleep, he was immediately awaked tormented by frightful dreams, or threatened with suffocation; nor could he remain two succeeding minutes in the same situation. The evening preceding his death, his agitation and anguish was inexpressible; I never before witnessed such suffering. His expectoration was black, and his urine also; his pulse was now unequal and small; the extremities of his fingers and toes were livid; he fell occasionally into a state of lipothymia, and asked for opium to relieve his pains. On the 30th of April he died.

*Dissection.*—The chest being opened, I found the pericardium dilated, thinner than usual, and filled with black blood; the heart very large, and its vessels distended with blood. The ventricles contained a large quantity of polypous concretions. Their parieties were thick, and not unhealthy; the right auricle very thin, and twice its natural size, had a small perforation in it, through which doubtless the blood had escaped into the pericardium; the left was also dilated, though not to the same extent; the greatest part of the septum ventriculorum was destroyed; the sigmoid valve was ossified; the right lung loaded with black blood, which in some parts was extravasated into its substance; the left lung was less disorganized; no effusion was found in the chest.

